# Background matching in the brown shrimp *Crangon crangon*: adaptive camouflage and behavioural-plasticity

**DOI:** 10.1038/s41598-018-21412-y

**Published:** 2018-02-19

**Authors:** Andjin Siegenthaler, Alexander Mastin, Clément Dufaut, Debapriya Mondal, Chiara Benvenuto

**Affiliations:** Ecosystems and Environment Research Centre, School of Environment and Life Sciences, University of Salford, Salford, M5 4WT UK

## Abstract

A combination of burrowing behaviour and very efficient background matching makes the brown shrimp *Crangon crangon* almost invisible to potential predators and prey. This raises questions on how shrimp succeed in concealing themselves in the heterogeneous and dynamic estuarine habitats they inhabit and what type of environmental variables and behavioural factors affect their colour change abilities. Using a series of behavioural experiments, we show that the brown shrimp is capable of repeated fast colour adaptations (20% change in dark pigment cover within one hour) and that its background matching ability is mainly influenced by illumination and sediment colour. Novel insights are provided on the occurrence of non-adaptive (possibly stress) responses to background changes after long-time exposure to a constant background colour or during unfavourable conditions for burying. Shrimp showed high levels of intra- and inter-individual variation, demonstrating a complex balance between behavioural-plasticity and environmental adaptation. As such, the study of crustacean colour changes represents a valuable opportunity to investigate colour adaptations in dynamic habitats and can help us to identify the mayor environmental and behavioural factors influencing the evolution of animal background matching.

## Introduction

The requirement of preys to avoid detection by their predators has led to the evolution of a wide range of strategies for animals to blend in their environment. Among crustaceans, some species are transparent^1^ and thus almost invisible, others allow the growth of epiphytes on their carapace^2^ or match their colour with the background, to conceal themselves^3–6^. Indeed, the ability to rapidly change colour is a common strategy employed by many animals to tune and adjust their camouflage abilities to heterogeneous environments^7–11^, as well as to communicate with conspecifics, thermoregulate and gather protection from ultraviolet (UV) light^7,12,13^. Colour adaptations represent a complex and multifaceted topic which connects environmental factors, animal behaviour, visual perception and cell physiology^11,14^. The assessment of how these factors are interlinked^15^ and contribute to the animal’s camouflage strategy is essential to understand the evolution of colour change and background matching in animals.

**Figure 1:**
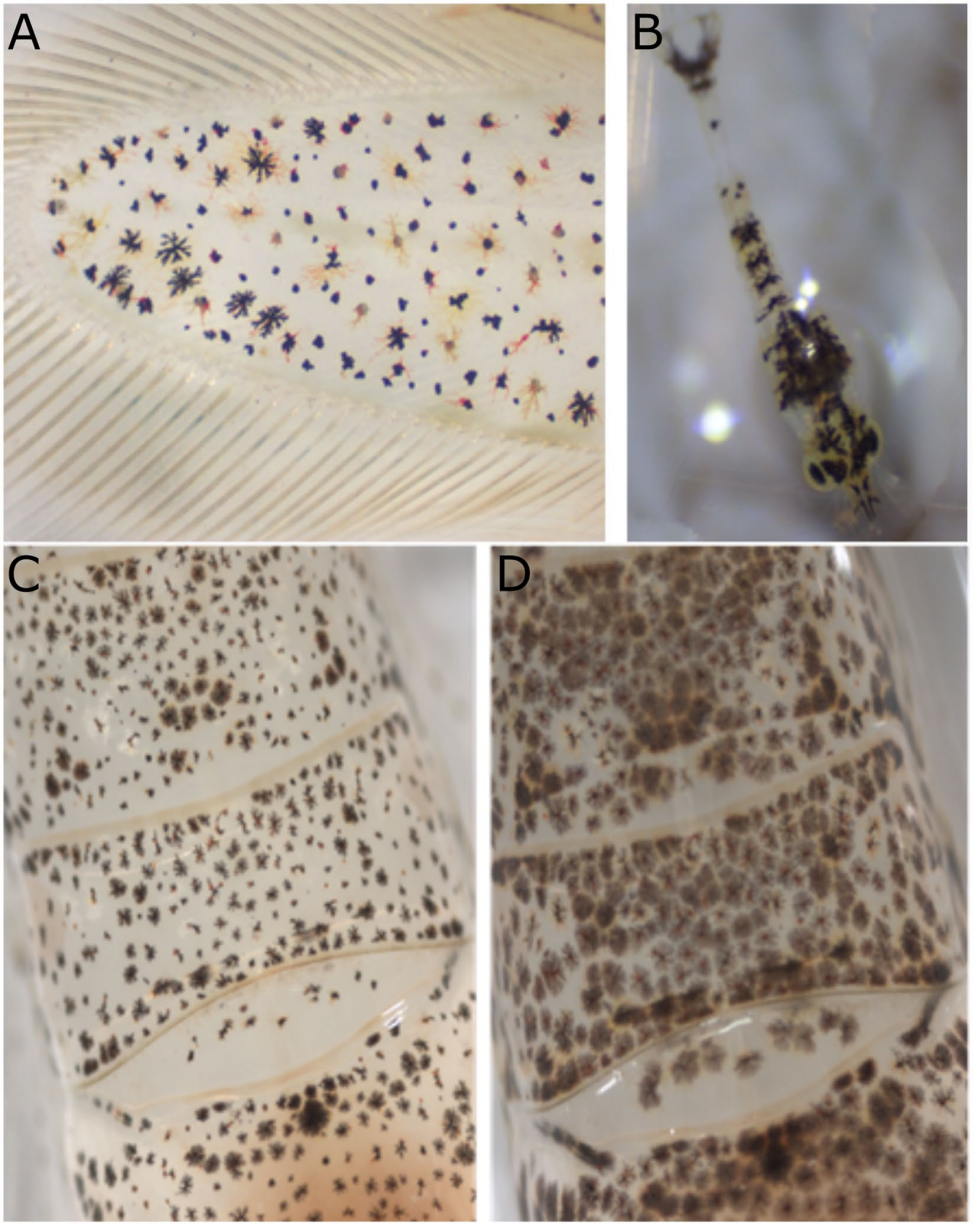
*Crangon* colour. Chromatosome pigments in exopod (part of the tail fan) of *Crangon crangon* (**A**); transparent larva of *C. crangon* with visible chromatosomes (**B**); physiological variation in *C. crangon* colouration in response to different coloured background: the same individual was photographed after having been placed on white (**C**) or black (**D**) backgrounds.

Crustaceans can change colour morphologically by the anabolism and catabolism of colour components (e.g., pigments; in days to months) or physiologically by rapid changes (in milliseconds to hours) in the distribution of pigments, microstructures or by changing the refractive index of layers in their integument^7^. Physiological colour changes by pigment migration within chromatophores (specialised cells containing pigmented organelles which can be dispersed or concentrated^16–18^; Fig. 1) are found in a variety of taxa^7^ and are well-studied at the physiological level^19–21^. Evidence on how colour changes fit in an ecological or evolutionary context is however still limited^7,9,11^.

Many crustaceans live in heterogeneous and dynamic intertidal systems in which biotic and abiotic factors vary over multiple spatial and temporal scales. A variety of these factors have the potential to influence camouflage strategies: the evolution and adaptive function of crustacean background matching should be partly driven by environmental variability^7,11–13^. Having a degree of flexibility in camouflage strategies is thus advantageous in these heterogeneous environments due to a continuous trade-off between conspicuousness and concealment^11,14,22,23^. Several environmental factors, such as temperature, tide levels, background, circadian rhythm and predation^7,24–27^ have been studied in a few crustacean species including fiddler crabs^24,28^, crabs^29,30^ and the shrimp *Hippolyte obliquimanus*^31^. Integrated approaches testing multiple environmental variables acting on other crustacean species are, however, rare.

*Crangon crangon* L. (Decapoda: Caridea) is a key species in European waters and an important target for fisheries^32–36^. Colour change is observed in the adults of this benthic shrimp^6^ (Fig. 1), which is surprising considering its lifestyle, with animals often found buried into the sediment, only eyes and antennae visible^37^. Its chromatophore system is well studied and was one of the earliest models of endocrine regulation of chromatophores^6,17,38–41^. Chromatophores are not individually distinguishable in *C. crangon*, but are combined (with multiple chromatophores of similar or dissimilar pigments) in a structure called the chromatosome^17^. Larvae are pelagic and after five weeks in the water column, post-larvae settle in shallow waters in estuaries^32,35^. Chromatosomes are also found in the transparent larvae (Fig. 1B), but they are more probably used for thermoregulation and UV protection at this stage (see below).

**Figure 2:**
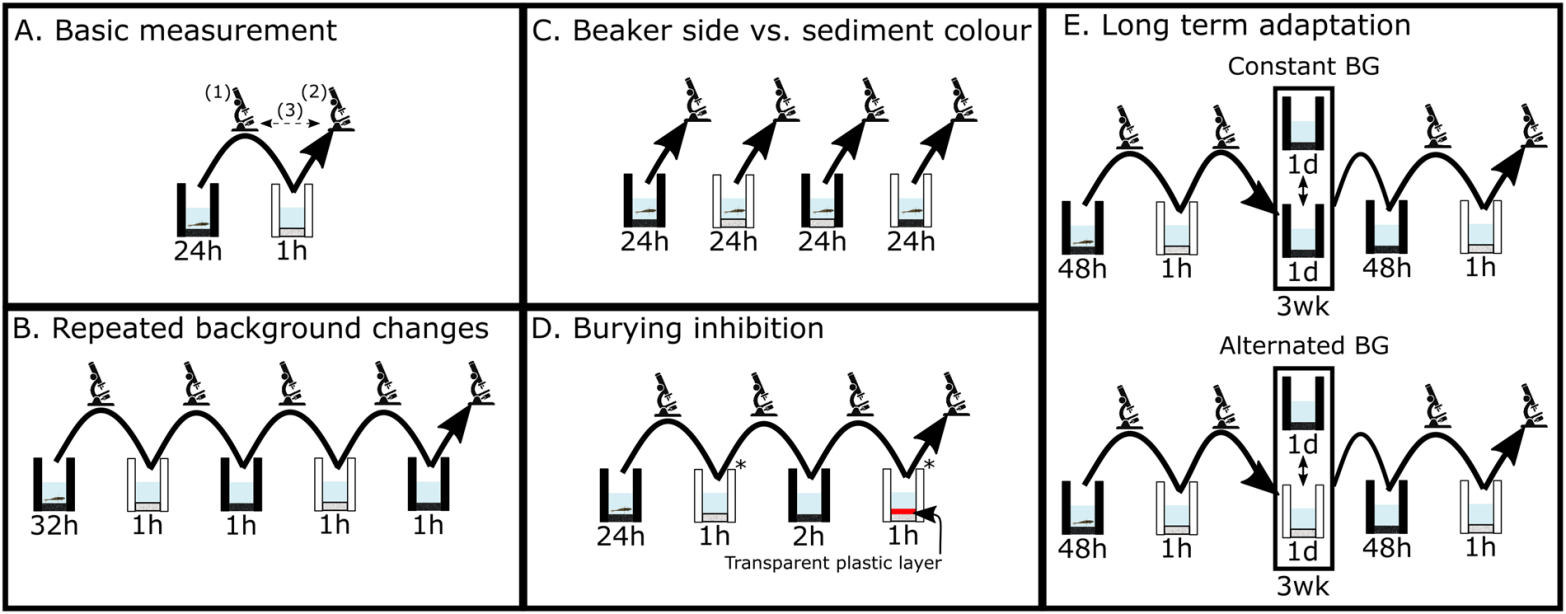
Schematic overview of the different protocols to determine the factors involved in *Crangon crangon* background matching. Arrows indicate shrimp being moved between beakers with different backgrounds, or to a dissecting microscope (microscope symbol) for pigment coverage measurements. Black and white colours indicate the colour of the sediment (bottom) and sides of the beaker. Time spent in each beaker is given below the beaker symbol. Repeated measurements with the same shrimp are indicated with continuous arrows. (1) First photo, (2) second photo, (3) colour change, * order randomly determined.

Seasonal migration to and from offshore mating areas characterize *C. crangon*’s life cycle^32,35^. Parallel to this broad seasonal and spatial variation, juveniles and adults experience local smaller scale variations in estuarine environment. Indeed, habitat characteristics such as illumination, presence or absence of vegetation, sediment colour and sediment composition vary frequently and sometimes unpredictably over space and time in intertidal areas. Biodiversity thus increases along this “gradient of structural complexity”^42^ due to habitat selectivity or ability of species to locally adapt to patchy/mosaic habitats. This study aimed to assess the effects of spatial and temporal environmental heterogeneity on the colour changing ability of *C. crangon*. Multiple behavioural lab experiments (Fig. 2) were conducted to analyse the influence of variation in illumination (over a day-night cycle), sediment colour and ease of burying on the background matching ability of this species, to gather a clearer understanding of the variability of colour adaptation in crustaceans.

## Results

### Biorhythm

**Figure 3:**
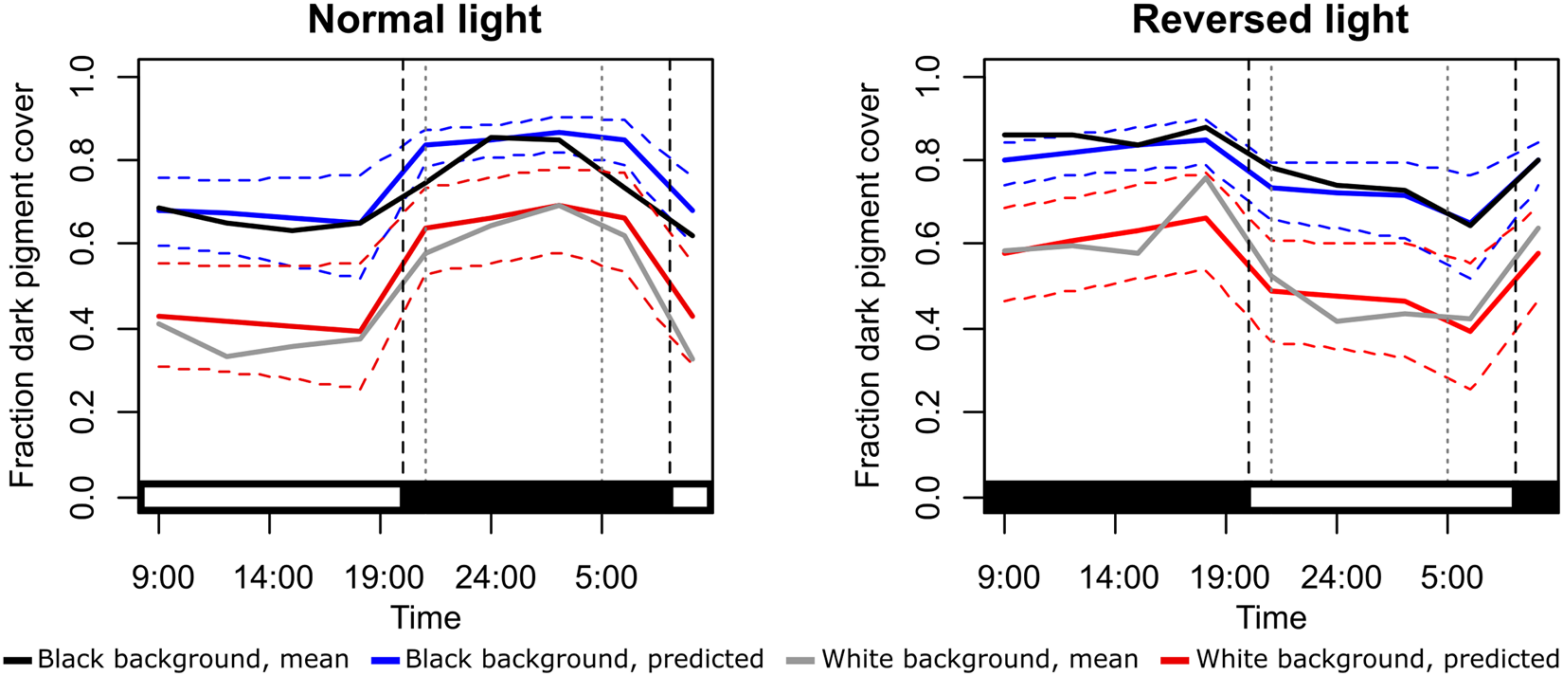
Effect of background colour and light on *Crangon crangon* mean dark pigment cover over a day-night cycle. The illumination regime is indicated with a black/white bar at the bottom of each graph. Solid lines: mean and predicted values on black and white backgrounds; dashed horizontal lines: confidence interval predictions; black dashed vertical lines: moment of light switch; period between grey dashed vertical lines: no day light. Measurements were made every 3 hours. N = 20 per treatment.

Model selection based on lowest Akaike information criterion (AIC) resulted in a final model with sediment colour and daylight as independent effects and an interaction term between artificial illumination and time since the artificial light status changed (TLC) (Fig. 3; Table S1). The model suggests that *C. crangon* are significantly (Z = −6.07; P < 0.001) paler (lower Pigment Cover, PiC) on a white background than on a black background (independently of the presence of light or time of the day) and significantly (Z = −5.38; P < 0.001) darker during the night than during the day (independently of the sediment colour and the presence of light). The interaction between artificial illumination and TLC can be interpreted as a significant (Z = −3.18; P = 0.001) progressive darkening of *C. crangon* after the artificial light was turned off. Plots of individual shrimp showed large intra- and inter-individual variation in pigment cover over a day-night cycle (Supplementary Fig. S1).

### Repeated background changes

**Figure 4:**
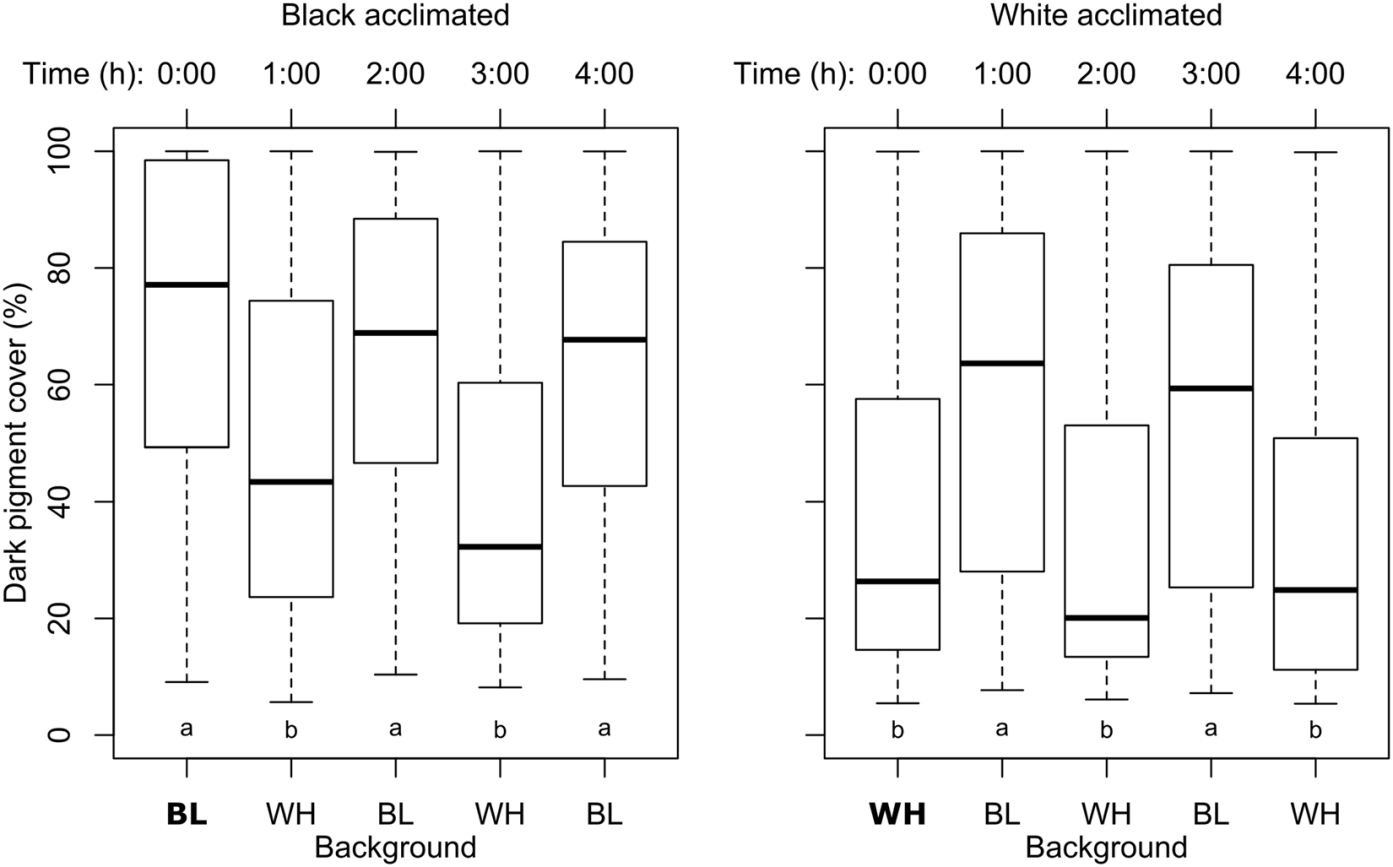
Box-and-whisker plots of dark pigment cover (%) during repeated shifts of *Crangon crangon* (N = 33) between black (BL) and white (WH) backgrounds. First measurement (in bold) was performed after 24 h acclimation and all subsequent measurements after 1 hour permanence on the respective background. Time: number of hours after acclimation. Different letters indicate groups that are significantly different (P < 0.01) based on Dunn-Bonferroni corrected post-hoc analyses.

*Crangon crangon* were significantly darker (higher PiC) when acclimated for 24 hours in black beakers than when acclimated in white beakers (Time 0:00, Fig. 4; Wilcoxon Test; N = 33, Z = −4.565, P < 0.001). Shrimp that were repeatedly transferred between black and white beakers (Fig. 2B) showed a consistent significant higher PiC in black beakers compared to white beakers, independent of the colour they were acclimated in (Fig. 4; Friedman Test with Bonferroni post-hoc correction: black acclimated: N = 33, χ^2^ = 43.32, P < 0.001; White acclimated: N = 31, χ^2^ = 58.10, P < 0.001). The specimens showed a consistent median change of 15% (mean: 20%) when moved between different backgrounds, independently of background colour (only the sign changed between positive and negative; Friedman Test on absolute values: N = 31, χ^2^ = 4.085, P = 0.770). Plots of individual shrimp showed large intra- and inter-individual variation in pigment cover (Supplementary Fig. S2) and a two-way ANOVA showed a significant influence of shrimp ID (Df = 30, F = 5.88, P < 0.001) and no influence of sediment colour (Df = 1, F = 0.351, P = 0.554) and acclimation  (Df = 1, F = 0.786, P = 0.376) on absolute colour change (%).

### Sediment vs. beaker colour

**Figure 5:**
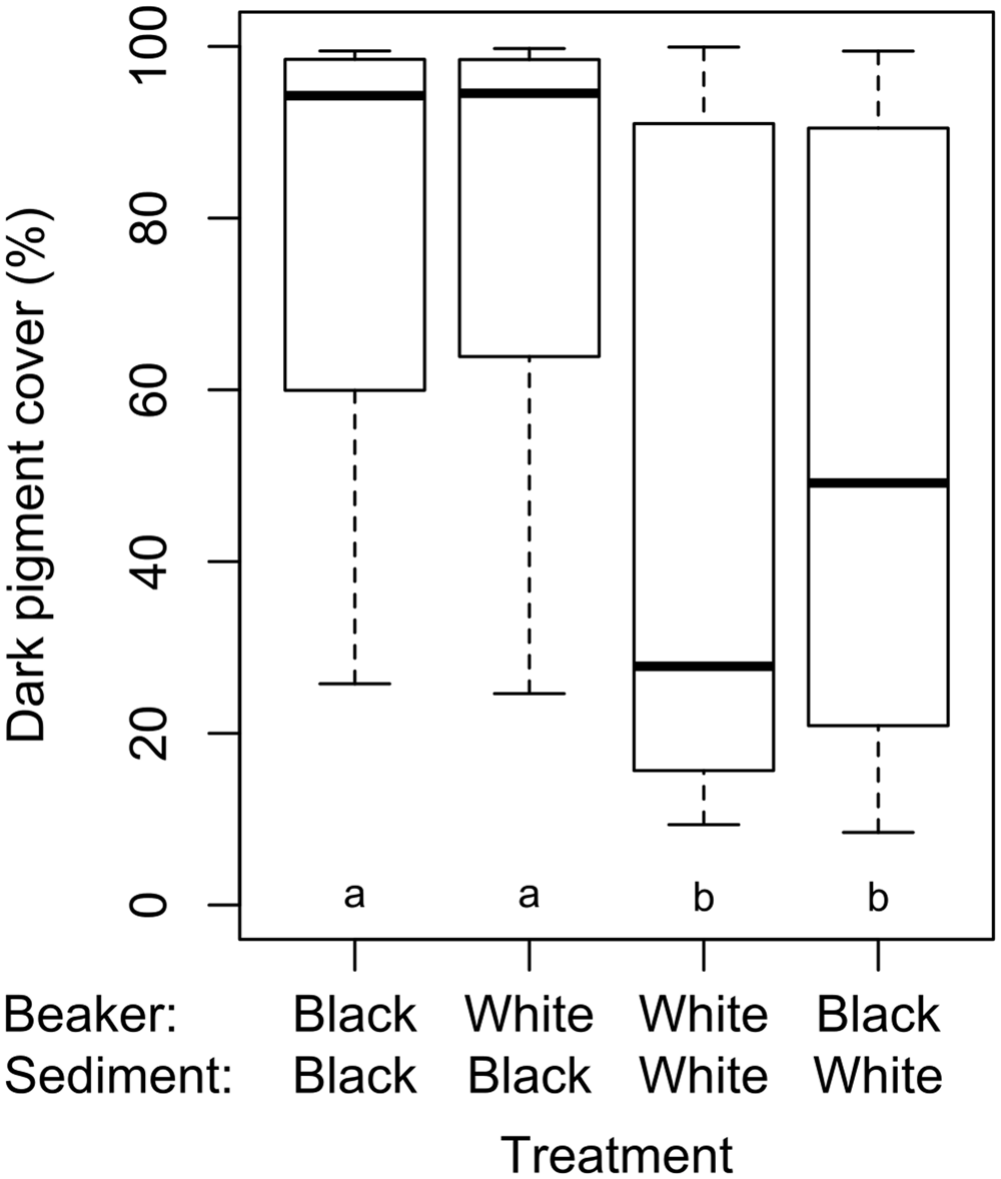
Box-and-whisker plots of dark pigment cover (%) of *Crangon crangon* (N = 32) kept for 24 h in beakers with different combinations of sediment and beaker colours. Different letters indicate groups that are significantly different (P < 0.01) based on Dunn-Bonferroni corrected post-hoc analyses.

Shrimp kept for a full day-night cycle in beakers with different combinations of sediment (black or white) and side colouration (black or white beaker; Fig. 2C) showed significant differences in PiC (Friedman Test: N = 32, χ^2^ = 22.9, P < 0.001). Dunn-Bonferroni corrected pairwise comparisons showed that PiC varied significantly with sediment colour but not with beaker colour (Fig. 5).

### Burying inhibition

**Figure 6:**
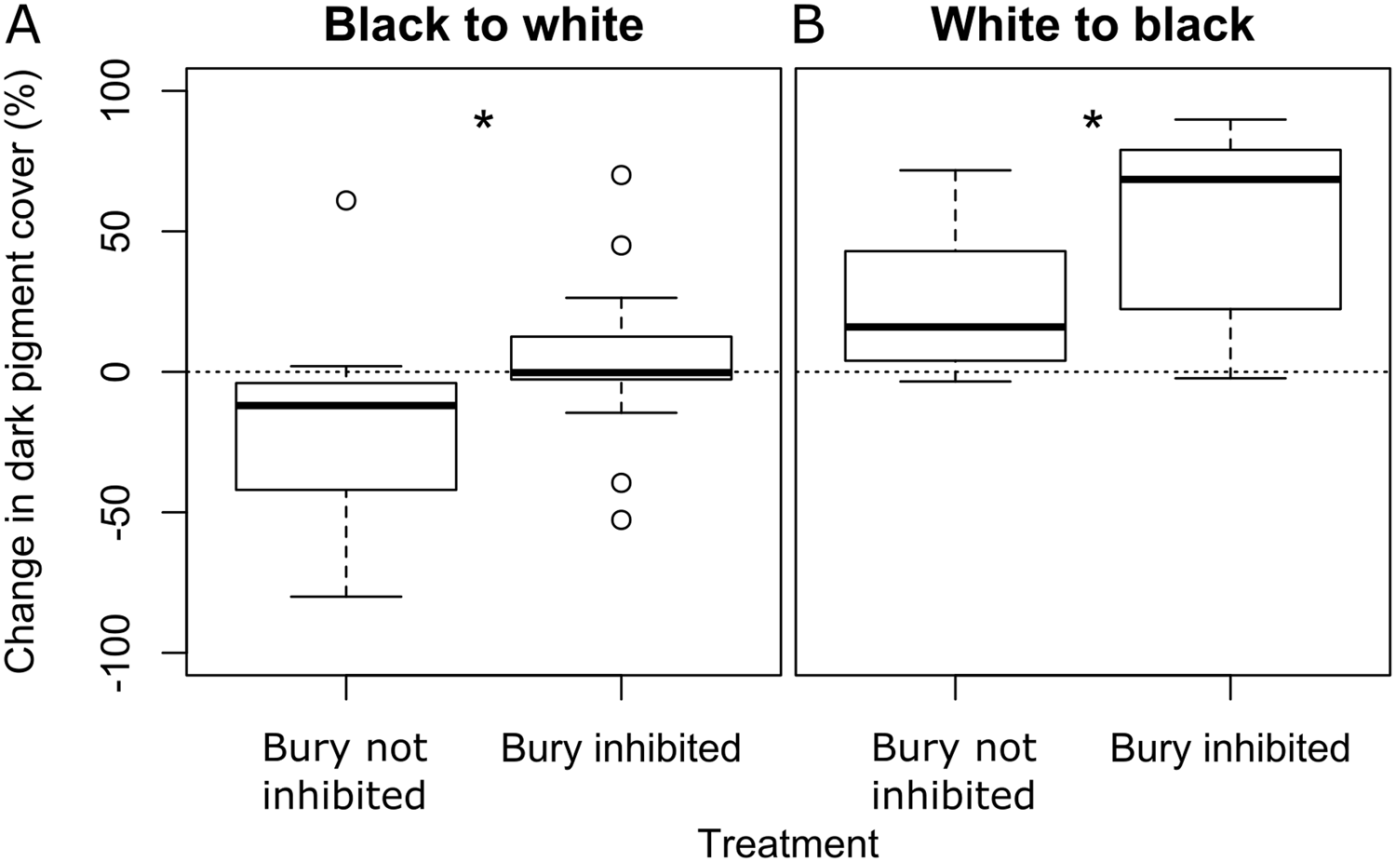
Effect of burying inhibition on *Crangon crangon* colour change. Box-and-whisker plots show the change in dark pigment cover within one hour of shrimp transferred to the opposite coloured background. (**A**) Shrimp were tested after acclimation on a black background and after being kept for one hour on a white background (N = 25). (**B**) Shrimp were tested after acclimation on a white background and after being kept for one hour on a black background (N = 22). The dashed line represents the level of no change. *P < 0.05. Change in pigment cover was calculated from PiC values shown in Figure S3.

Preventing *C. crangon* from burying (Fig. 2D) resulted in darker shrimp, regardless of the background colour. Shrimp that were transferred from black to white backgrounds did not change colour as much when their burying behaviour was inhibited compared to shrimp that could bury (Fig. 6A; Wilcoxon Test; N = 25, Z = −2.381, P = 0.017) resulting in no median difference in PiC before and after the transfer (Supplementary Fig. S3; median PiC BL = 92%; median PiC WH = 96%; Wilcoxon Test; N = 25, Z = −0.748 P = 0.455). On the contrary, *C. crangon* transferred from white to black backgrounds showed a significant larger median change in colour when their burying behaviour was inhibited than when they could bury (Fig. 6B; Wilcoxon Signed Ranks Test; N = 22, Z = −3.750, P < 0.001). A total of 10 black acclimated and 8 white acclimated specimens were excluded from analysis because they did not bury when allowed. The acclimation time (24 hours vs. 2 hours; Fig. 2D) did not influence *C. crangon* PiC values during the initial measurements (Wilcoxon Test: WH: N = 32, Z = −1.215, P = 0.224; BL: N = 31, Z = −1.568, P = 0.117).

### Long term adaptation

**Figure 7:**
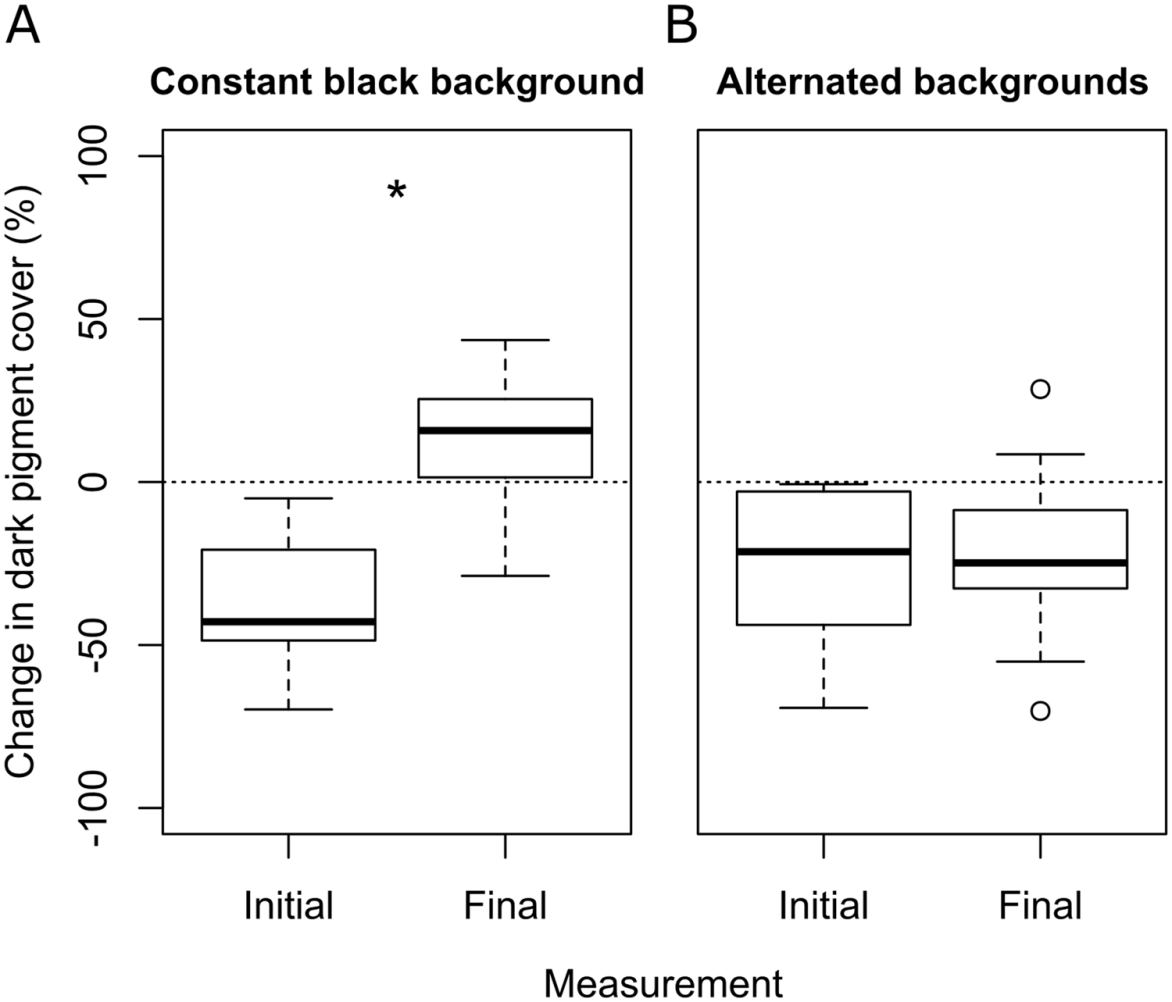
Effect of long term background adaptation on *Crangon crangon* colour changes. Box-and-whisker plots show the change in dark pigment cover within one hour of shrimp transferred from a black to a white background. Shrimp were first tested at day 0 (initial), than exposed to either (**A**) a constant black background (N = 11) or (**B**) daily alternating black and white backgrounds (N = 15), and tested a second time at day 21 (Final). *P < 0.05.

Long term exposure to a single background colour (Fig. 2E) had a negative impact on the ability of *C. crangon* to change colour. When transferred from black to white backgrounds, shrimp that were exposed exclusively to black backgrounds for 21 days showed a decreased ability to match the white background compared to their initial ability, as measured at the beginning of the experiment (day 0; Fig. 7A; Wilcoxon Test: N = 11, Z = −2.934, P < 0.003; Supplementary Fig. S4; median PiC Black = 65%; median PiC White = 89%; Wilcoxon Test: N = 11, Z = −1.778, P = 0.075). On the contrary, shrimp that were exposed to daily switches between backgrounds maintained their ability to match the colour of the background (Supplementary Fig. S4; median PiC Black = 65%; median PiC White = 37%; Wilcoxon Test: N = 15, Z = −2.613, P = 0.009) and showed no difference in colour change between the initial and final measurements (Fig. 7B; Wilcoxon Test: N = 15, Z = −0.227, P = 0.820). A total of four shrimp died during this experiment and were excluded from analysis.

## Discussion

Background adaptation of crustaceans is a complex process, influenced by multiple parameters^5^, intrinsic (e.g., sex^6,43^, moult stage^6^, stress^25^) and extrinsic (e.g., spatial and temporal environmental factors, including temperature^24,26,30^, UV radiation^12^, circadian rhythm^12^, tides, predation^27,44^). Here, we have focused on variation in illumination and sediment colour on the colour change abilities of *C. crangon*.

The overall colour of *C. crangon* showed a clear rhythm, being darker during the night and paler during the day. Other crustaceans also show similar colour patterns regulated by a circadian rhythm, as the horned ghost crab *Ocypode ceratophthalmus*^30^, even though in the majority of the species the pattern is inversed (darker during the day than at night)^5,43^, as in the fiddler crab *Uca panacea*^12,30^. This nocturnal colouration was already observed at the end of the 1800 century in *Hippolyte varians*^45^. Darker colouration during the day might reflect the use of chromatophore expansion as protection to UV light rather than a strategy to match the background. Indeed, also in *C. crangon*, there is a primary reaction^6^ to light (with expansion of black pigments in the chromatophores) in response to increased intensity of incident light. In this experiment though, light intensity was not changed. Only absence-presence of light (mimicking night-day variations) was tested. These rhythms observed between absence-presence of light might facilitate either concealment/camouflage, energy saving, thermoregulation, UV protection, or a combination of factors, which might change with life history stages and sex^3,6,30,46–48^. In our study, we recorded a darkening of the colour during the night independently of the illumination provided to the shrimp, but we cannot definitely call this a circadian rhythm since no experiments were conducted in constant darkness as would be required to check the endogenous process linked to colour change^49^. Background matching during the full day-night cycle may enable *C. crangon* to camouflage itself during low light conditions, when shrimp are more active^33,50,51^ and possibly more prone to predation. Thermoregulation and UV protection are, on the other hand, likely of secondary importance in this case, since adult *C. crangon* live for the majority of the time submerged in temperate and turbid estuaries, almost completely buried in the sediment^30,33,35,52,53^. They might be more important at the pelagic larval stage (Fig. 1B), as reported in other decapods^54,55^.

Background matching is an important camouflage strategy of *C. crangon*, as already described since the early 1900s^41^. Changes in background colouration resulted, on average, in a 20% change in dark pigment cover within one hour, this change being constant over repeated background switches. A constant colour change rate is in contrast to the behaviour of several species of flatfish, where melanophore response rates increase during repeated background switches^56,57^. Variation between individual *C. crangon* was high, which was compensated by repeated measurements on the same animal. Comparable rates of changes are observed in several other species of decapods^7^, but some crustaceans show, in contrast to *C. crangon*, differences in the rate of change between pigment dispersion and concentration^49,58^. C*rangon handi* is, for example, more successful in adapting to dark- than light-coloured substrates^59^, while the opposite has been recorded in the ghost crab *O. ceratophthalmus*^30^. Differences in the rate of dispersion between different pigments are known for *C. crangon*^6^, but were not measured during this study. The costs of pigment dispersion and concentration are also not fully understood^3,7,13,55,60^.

**Figure 8:**
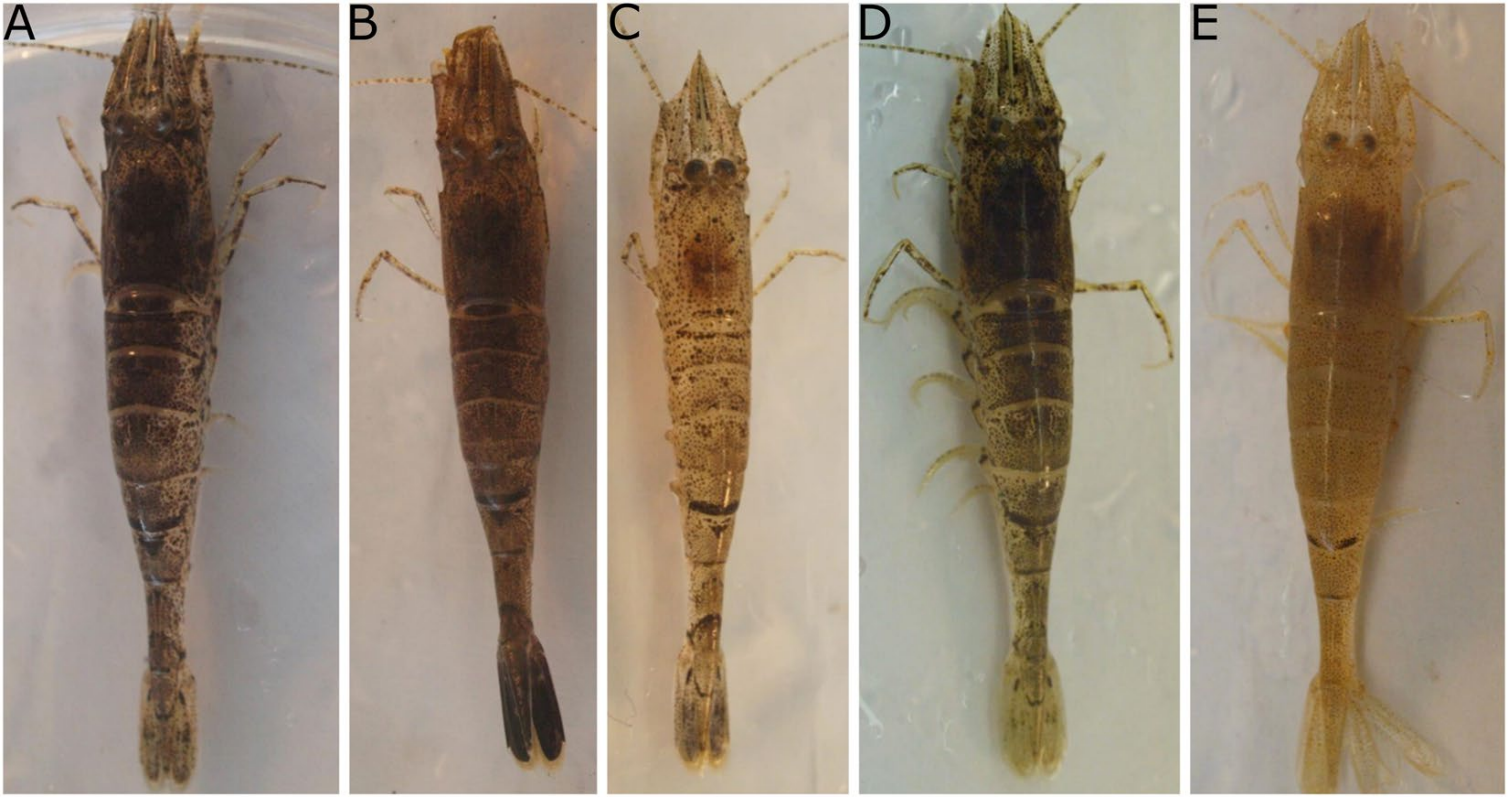
Examples of *Crangon crangon*’s variation in natural colouration. Specimens were caught in the beach in front of Dale, Pembrokeshire, in the Cleddau Ddu estuary. (**A**–**C**) shrimp collected from muddy-sandy substrates; (**D**) shrimp collected from algae-dominated sand; (**E**) shrimp hold in a yellow bucket (notice the high spread of yellow pigments in chromatophores).

Sediment colour is the main factor determining *C. crangon* colouration and in the field shrimp with naturally variable occurring colours have been observed (Fig. 8). Colours of structures above the sediment level (mimicked by the colour of the side of experimental beakers) did not have an effect on the shrimp’s colour. This is not the case in other species with life-styles not so linked to the sediment: the colour of *Lysmata boggessi*, kept in the lab, for instance, is affected by the radiation reflected from the side colour of tanks^61^. The side colour of rearing tanks also influences larval survival and productivity in *Macrobrachium amazonicum*^62^. The ability to match the colour of the sediment provides a camouflage advantage when shrimp emerge on light sediment besides darker structures, e.g., rocks. Background adaptation depends on the ratio of the light reflected from the environment to incident light^24,63–65^. Due to its low profile, the majority of the light conveyed in *C. crangon*’s eyes will be reflected from the sediment, explaining the low relevance of vision of other nearby objects for background matching. This also explains the good matching colour of buried individuals (as the eyes receive the stimulus even when the body of the shrimp is covered by the substrate). Keeping its eyes above the sediment^37^ allows the shrimp to continuously respond to light stimuli while buried, avoiding conspicuousness when emerging from the sediment^30^.

Patchiness in sediment colouration and ease of burying (e.g., due to sediment compactness) might also influence *C. crangon* colour. Long-time exposure to constant dark sediments resulted in darker shrimps during colour-change experiments. Background matching mechanisms likely vary among time and spatial scales^22^. Individuals inhabiting environments with more heterogeneous/patchy colours might be better adapted to reply to background changes than individuals that are exposed to a single background for a prolonged period of time^63,65^. Chromatic adaptations to a single background colour could further be influenced by the behaviour of the species, being more pronounced in species inhabiting in more consistent environment compared to others, such as *Carcinus maenas*^46^, exposed to multiple habitats due to active daily movements. Indeed, active responses to visual predators in crabs (habitat choice when predatory clues are detected)^44^ can be combined with colour morphs, especially in juveniles^27^. In the brown shrimp, juveniles do not present variable colour morphs, as the overall cryptic strategy is to burrow and match the substrate. Inhibition in *C. crangon* burying behaviour also resulted in darker shrimps, independently of the sediment colour. Darkening of the shrimp might in this case represent a “visual stress sign” to unfavourable environmental conditions. Studies on multiple species of fish have shown a link between pigment regulation and stress responses for melanin dispersing hormones^66–68^ and several invertebrate species are known to become darker or red when subjected to handling stress^25,60,69,70^, including the fiddler crab *Uca vomeris*^28^. This possible link between environmental stressors and background adaptations represents a very interesting topic for further research, as behavioural responses can be early sign of stressors in animals. Even in lab experiments, responses to stressors should be taken into account, especially considering that essential works on *C. crangon* colour changes have been performed in aquaria without a sediment layer^6,41^, which is known to be a major stressor to the shrimp^71,72^.

Habitat characteristics such as light, sediment colour and sediment compactness vary across spatial and temporal scales in heterogeneous and dynamic habitats as estuaries and can influence *C. crangon* colouration and background matching (Fig. 8). Our results confirm that this behaviour depends on an interlinked set of environmental and behavioural parameters^15,22^. The complexity of this behavioural response results in high inter- and intra-individual variation in background matching and mismatching, as has been observed in *Crangon* spp.^19,41^ and other crustaceans^3,22,30^. Phenotypic variations, resulting in well distinguished morphotypes are present in many crustaceans e.g., *H. obliquimanus*^31^ and could be produced by genetic polymorphism or plasticity. Such variability in colour is not restricted to crustaceans and can play a role in speciation in a variety of taxa^73^. In *C. crangon* specific colour morphs are not present, not even in the juvenile stages, as it occurs in other crustaceans^27^, rather a variety of responses to match the substrate across individuals as well as in the same individual when recorded for prolonged periods of time. These variations might combine a mix of adaptive and non-adaptive (including stress) responses^74^ and/or can make the overall populations more adapt to sudden changes (especially in a world where human impacts are sudden and unpredictable). Identifying individual variation and the mechanisms behind this variation is essential for assessing ecological and evolutionary processes^75^. Besides identifying possible drivers for inter- and intra-specific variation, studies on colour change also provide information on how animals adapt to different environments and on the relationship between visual perception and animal colouration^30^. The inter- and intra-specific variation recorded under controlled lab conditions is expected to be even amplified in the field. In those conditions, the main key ecological drivers influencing colour change will be acting concurrently. Once mechanisms and specific adaptations are uncovered and interpreted, lab experiments should be paralleled by field experiments. This study demonstrates that the ability of animals to change colour is a delicate balance of behavioural-plasticity and environmental adaptation at different spatial/temporal scales^29^. Failure to adapt to the right colour under the right circumstance influences survival^55,76,77^ so the prioritization of the factors influencing background matching should be under strong selection pressure. Taking into account the factors that play a role in determining animal colouration is, therefore, an essential step in the understanding of the evolution of animal colour change in heterogeneous and dynamic habitats.

## Methods

### Collection and maintenance of the test organisms

*Crangon crangon* were collected by push net (1.2 mm^2^ mesh size) in the Cleddau Ddu estuary (Lower Waterway) close to the town of Dale (Pembrokeshire, UK) on April 2015 and by bottom trawl (2 mm^2^ mesh size) in Morecambe Bay (Flookburgh, UK) on June 2015. The first area is characterized by fine/muddy sand with gravel/rocks and green and brown algae^78^, on which shrimp displayed quiet a variety of natural colours (Fig. 8) while in Morecambe bay the sediment at the sampling site (further away from the coast, reached at low tide) was characterized by more homogeneous brown mud. Shrimp were placed in aerated buckets and transported to the lab where they were acclimated in gently aerated 50 L glass aquaria with a ~1 cm thick layer of black, white (Pettex Roman Gravel) or yellow (ProRep Desert sand SPD005) sand for at least a week prior to any experiments. Artificial sea water (Aquarium Systems, Instant Ocean) was used, with salinity and temperature (mean ± SD) maintained at 24.2 ± 5.9 PSU and 16.9 ± 2.3 °C respectively. Captured *C. crangon* had a mean (±SD) total length (TL) of 50 ± 6 mm and a 5.5:1 Female:Male sex ratio. Prior to the experiments, to avoid cannibalism^79,80^, shrimp were moved to individual 2 L beakers (Ø: 13 cm) containing ~1 cm (0.1 dm^3^) of sediment and 500 ml of artificial sea water, gently aerated. Beakers were wrapped in white or black paper (called “black” (BL) or “white” (WH) beakers) matching the sediment colour contained at the bottom of the beaker (unless stated otherwise), to avoid any external visual influence. Shrimp were fed *ad libitum* with fish, three times a week and any leftover food was removed two hours after feeding. Water was partially changed once per week. All shrimp were kept under a 12 h:12 h artificial illumination regime (Sylvania T5 830 fluorescent tube; light on: 8:00–20:00) but natural light from windows was not blocked (influencing the light cycle to a minor extent).

### Colour measurements

Colour measurements were conducted following the protocol of Siegenthaler and colleagues^81^. Briefly, shrimp colouration was quantified as the percentage area covered by dark (black/brown) pigments^19,41^ on a section of the shrimp’s exopod of the tail fan^19^. Images of the exact centre (1 mm^2^) of the right tail fan were made on a white stage under a Leica S6D dissecting microscope (Illumination: two JANSJÖ led spotlights: 88 lm, 3000 Kelvin) and saved with Leica Application Suite v4.3.0. Dark pigment cover (PiC) was calculated using the colour threshold function default thresholding algorithm and minimum manual adaptations^81–83^ in ImageJ version 1.48^84^.

### Biorhythm

To test whether the colour of *C. crangon* was subjected to a biorhythm, PiC was measured every three hours^12^ over the course of a full day-night cycle (starting at 09:00). Shrimp were randomly divided over black and white beakers and two different illumination treatments (“natural” and “reversed”). In the former treatment, beakers were kept under natural illumination, complemented by artificial illumination from 08:00 to 20:00; in the latter treatment beakers were kept in the dark with artificial light from 20:00 to 08:00. Temperature was maintained at 19.4 ± 1.1 °C during the day and 19.8 ± 0.6 °C at night. The shrimp were exposed for 84 hours to their assigned illumination regime, under the same conditions. Exposure to light during dark-period measurements was minimal (one minute required to take a photo). A total of 80 shrimp were tested over two day-night cycles.

### Background matching

A series of four experiments were conducted to test the effects of variation in sediment characteristics on *C. crangon*’s background matching ability (Fig. 2). For all experiments, shrimp were first acclimated in either black or white beakers and the initial PiC was estimated^81^. Colour change was measured before and one hour after moving shrimp to the opposite sediment colour (Fig. 2A). The effect of repeated background changes (Fig. 2B) was investigated by switching shrimp (N = 40) four times between black and white sediments (one hour time interval). The relative effects of colour of sediment and beaker sides (representing the surroundings above the sediment level) was assessed by varying both the colour of the sediment and beaker sides (Fig. 2C), with all shrimp exposed to all four treatments (on different days) to account for individual variation.

In all experiments, shrimp were allowed to bury, at least partially^37^, in sand. To test the effect of burying inhibition (Fig. 2D), shrimp (N = 32) were moved onto the opposite sediment colour for one hour and PiC was measured. These shrimp were then returned to the original coloured sediment for two hours before being placed again onto contrasting sediment for another hour. During the first or the last move, shrimp were placed in beakers with or without a plastic layer (burying prevented and control), in random order. Shrimp that did not bury when permitted were excluded from further analysis. In the repeated measurements and burying inhibition experiments (Fig. 2B,D), each shrimp was tested starting from both colours, on different days.

Finally, long-term adaptation of *C. crangon* to a single or varying background colour(s) was tested keeping shrimp constantly in black background (CST) compared with shrimp switched between backgrounds, alternating white and black backgrounds every other day (ALT; Fig. 2E). The PiC of shrimp (N = 15) before and one hour after movement from black to white sediment was assessed before and after three weeks of CST or ALT treatment. During this time, even CST shrimp were removed and replaced in their beakers to ensure equal levels of handling. To avoid any prior adaptation to black sediment, all shrimp were kept on yellow sediment for three weeks before the experiment started.

### Data analyses

Nonparametric tests (Mann-Whitney U, Wilcoxon Signed Rank and Friedman Tests) were applied to test for differences in PiC and the degree of colour change between treatments^85–87^. The degree of colour change was estimated as the difference in PiC values (Fig. 2A), with positive values indicating that the animal became darker and negative values indicating that the animal became paler. Shrimp that died during the experiments were excluded from analysis. R statistical software v.3.1.2^88^ was used for data analysis.

Biorhythm was analysed using the glmmADMB R package^89^ to create a generalised linear mixed beta regression model^81,90^. The fixed effects assessed were: sediment colour (WH, BL), presence of daylight (day: 05:00–21:00, night: 21:00–05:00), artificial illumination (on, off) and the time since the artificial light status changed (TLC). Shrimp ID was included as a random factor, in order to capture some of the autocorrelation in the model. A full model was constructed which contained interactions between all terms. Individual terms were removed from the model if their removal reduced the absolute value of the AIC by more than 2^91^.

### Ethics

This study and animal husbandry protocol used has been reviewed and approved by the Science & Technology Research Ethics Panel of the University of Salford (application STR1617-09) and adheres to the ASAB/ABS Guidelines for the Use of Animals in Research as well as the legal requirements of the United Kingdom.

### Data accessibility

The datasets supporting this article have been uploaded as part of the supplementary material (Supplementary data).

## Electronic supplementary material

Supplementary Material

Supplementary Dataset
