# Supplementary material for: Background matching in the brown shrimp Crangon crangon: adaptive camouflage and behavioural-plasticity
Source: Sci Rep. 2018 Feb 19;8:3292. doi: 10.1038/s41598-018-21412-y (PMC5818513; doi:10.1038/s41598-018-21412-y)
Supplement: Supplementary file 1 — Supplementary Material [file 41598_2018_21412_MOESM1_ESM.doc]

**Supplementary material**

This file contains supplementary figures and tables for:

**Background matching in the brown shrimp *Crangon crangon*: adaptive camouflage and behavioural-plasticity**

**Andjin Siegenthaler, Alexander Mastin, Clément Dufaut, Debapriya Mondal, Chiara Benvenuto**

**Table S1. Biorhythm model coefficient estimates. Coefficients estimates are shown for the model incorporating sediment colour (white vs. black), artificial illumination (on vs. off), presence of day light, time since change of the light regime (TLC) and the interaction between artificial Illumination and TLC.**

| **Factor** | **Coefficient (±SE)** | **z-value** | **P** |
| --- | --- | --- | --- |
| Intercept | 1.58 ± 0.15 | 10.70 | < 0.0001 |
| Illumination (on) | -0.56 ± 0.10 | -5.38 | < 0.0001 |
| Sediment colour (white) | -1.06 ± 0.17 | -6.07 | < 0.0001 |
| TLC | 0.04 ± 0.01 | 3.15 | 0.0016 |
| Daylight | -0.25 ± 0.06 | -3.86 | 0.0001 |
| Illumination (on) : TLC | -0.05 ± 0.02 | -3.18 | 0.0015 |


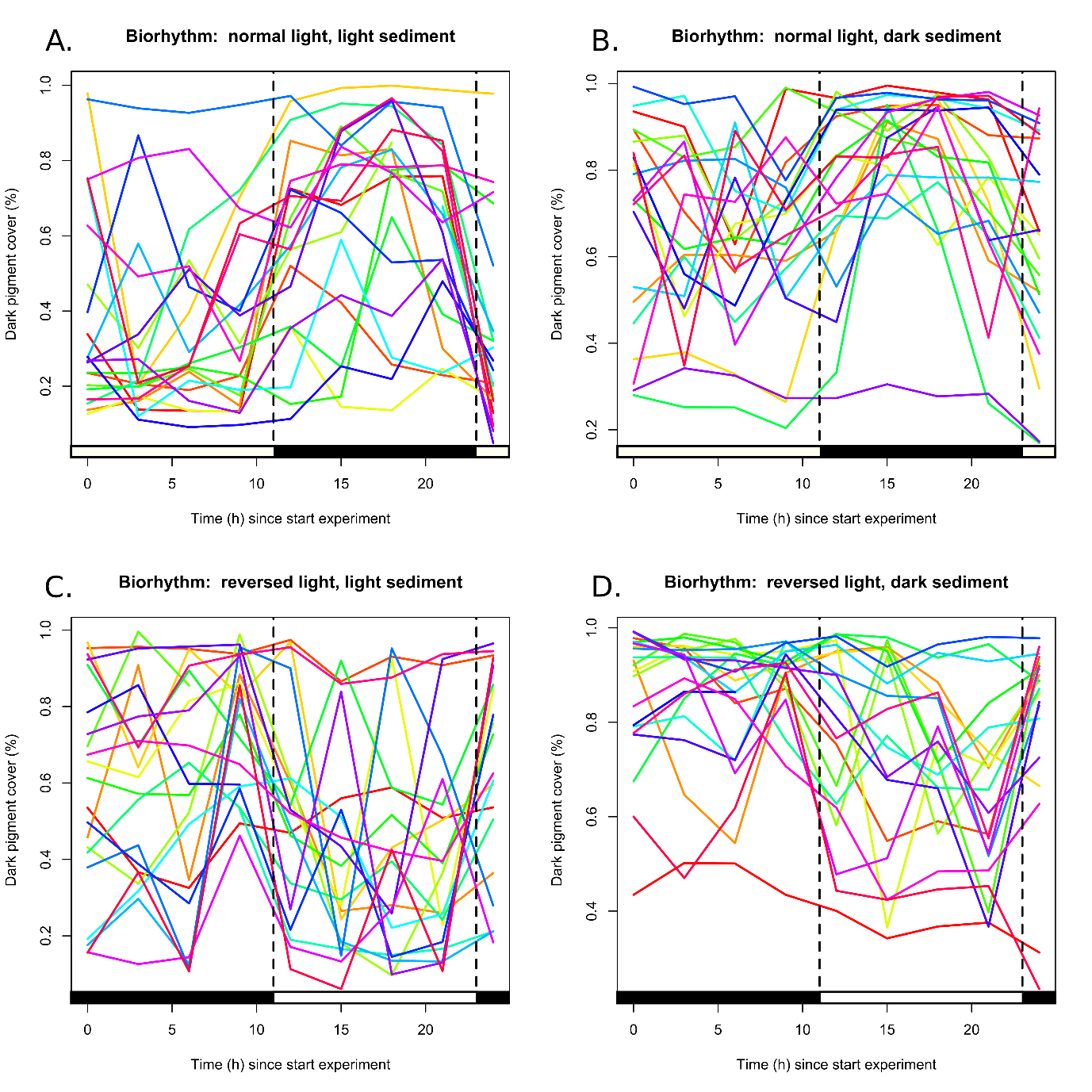


**Figure S1.** Effect of background colour and light on individual *C. crangon* dark pigment cover over a day-night cycle. The illumination regime is indicated with a black/white bar below each graph. Each line represents a different individual. Dashed vertical lines: light switch. Time = 0 is 9:00am.


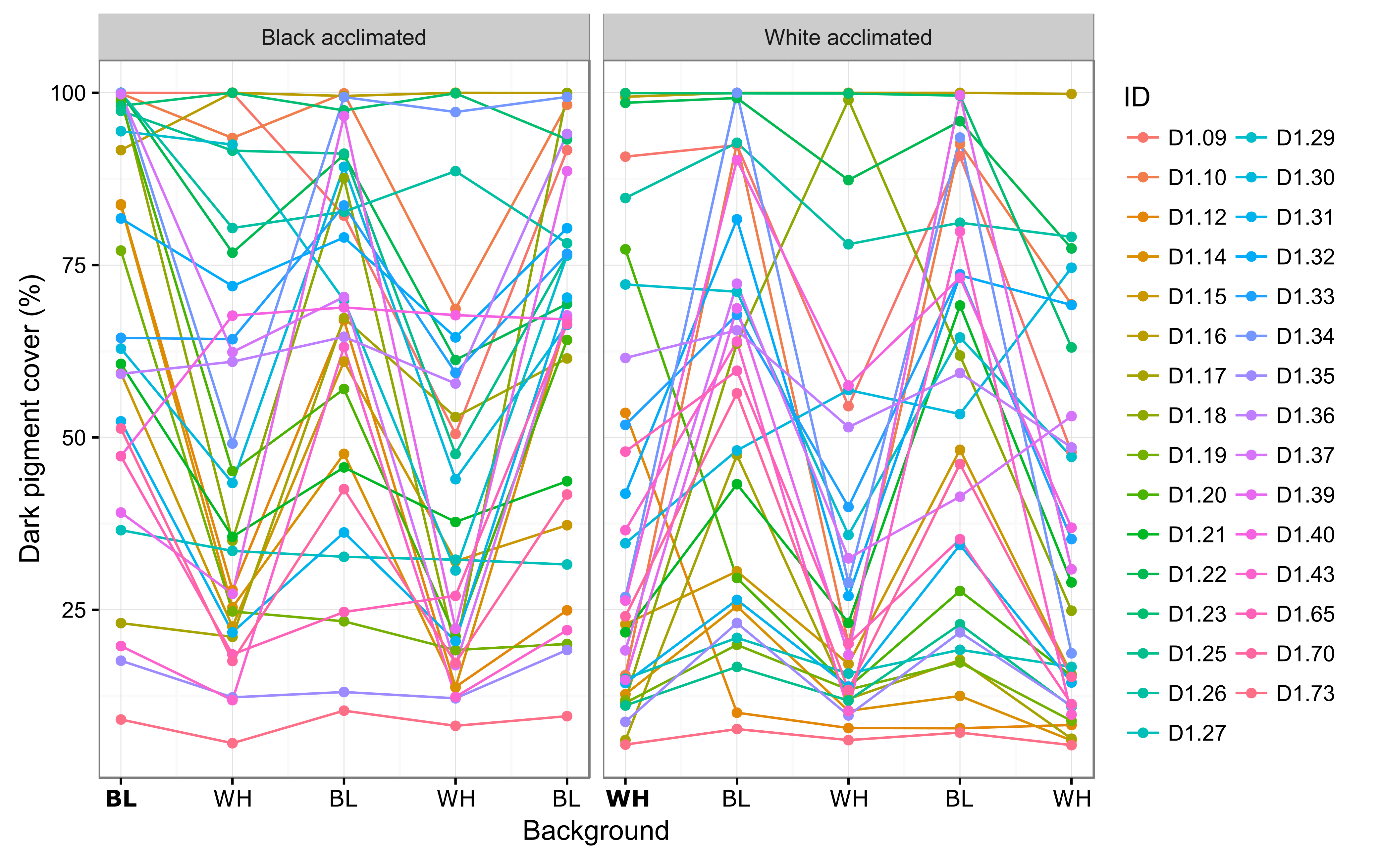


**Figure S2.** Dark pigment cover (%) of individual *C. crangon* (N = 33) during repeated shifts between black (BL) and white (WL) backgrounds. First measurement (in bold) was performed after 24h acclimation and all subsequent measurements after 1 hour permanence on the respective background. Each colour represents a different individual (ID).

**
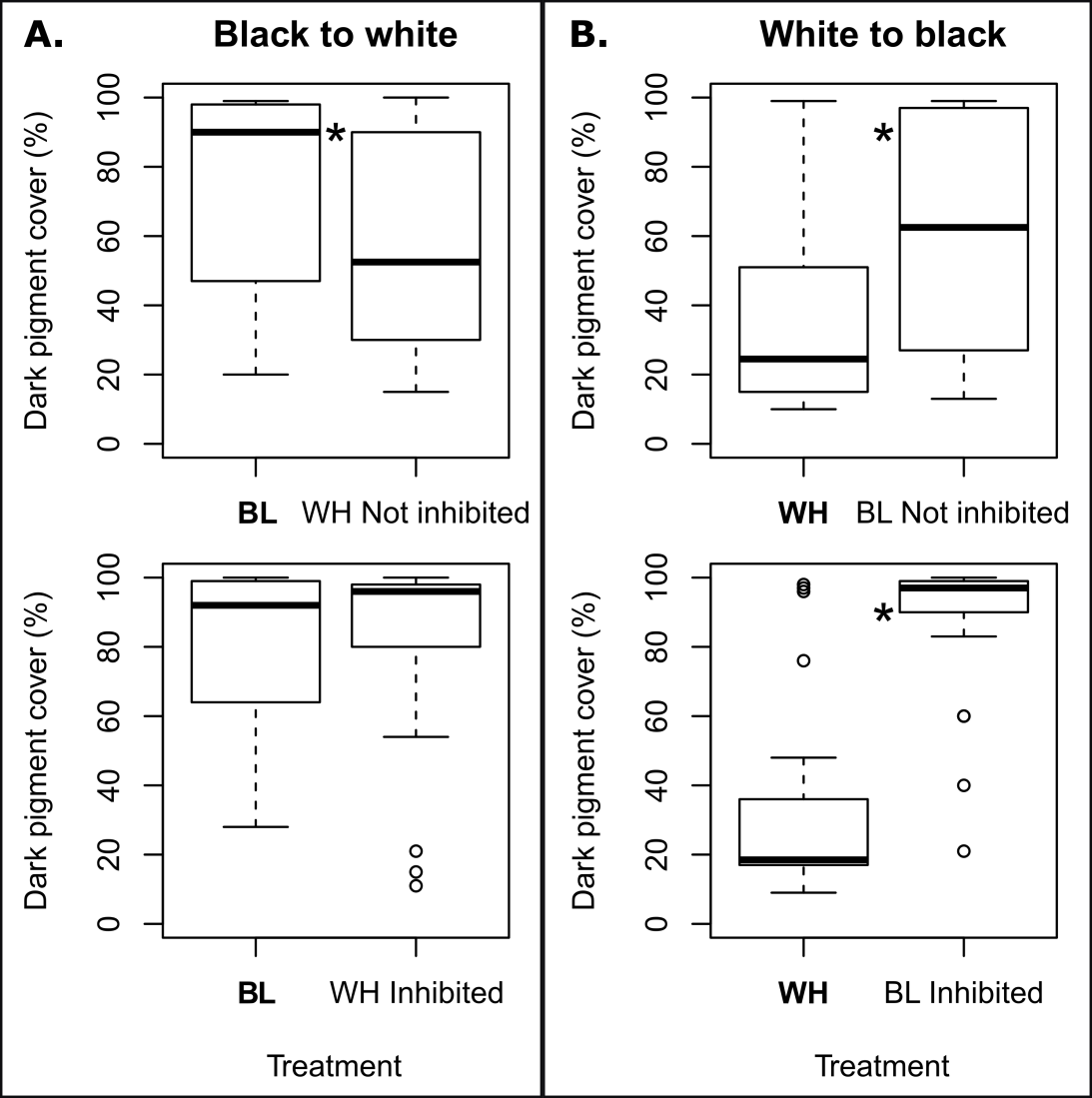
**

**Figure S3.** Box-and-whisker plots showing dark pigment cover (%) of *C. crangon* that were inhibited or not inhibited from burying on black (BL) or white (WH) sediment. A: Shrimp were acclimated on black sediment (bold) and moved to white one hour (N = 25). B: Shrimp were acclimated on white sediment (bold) and moved to black for one hour (N = 22). *: P < 0.05 (Wilcoxon Signed Rank Test).


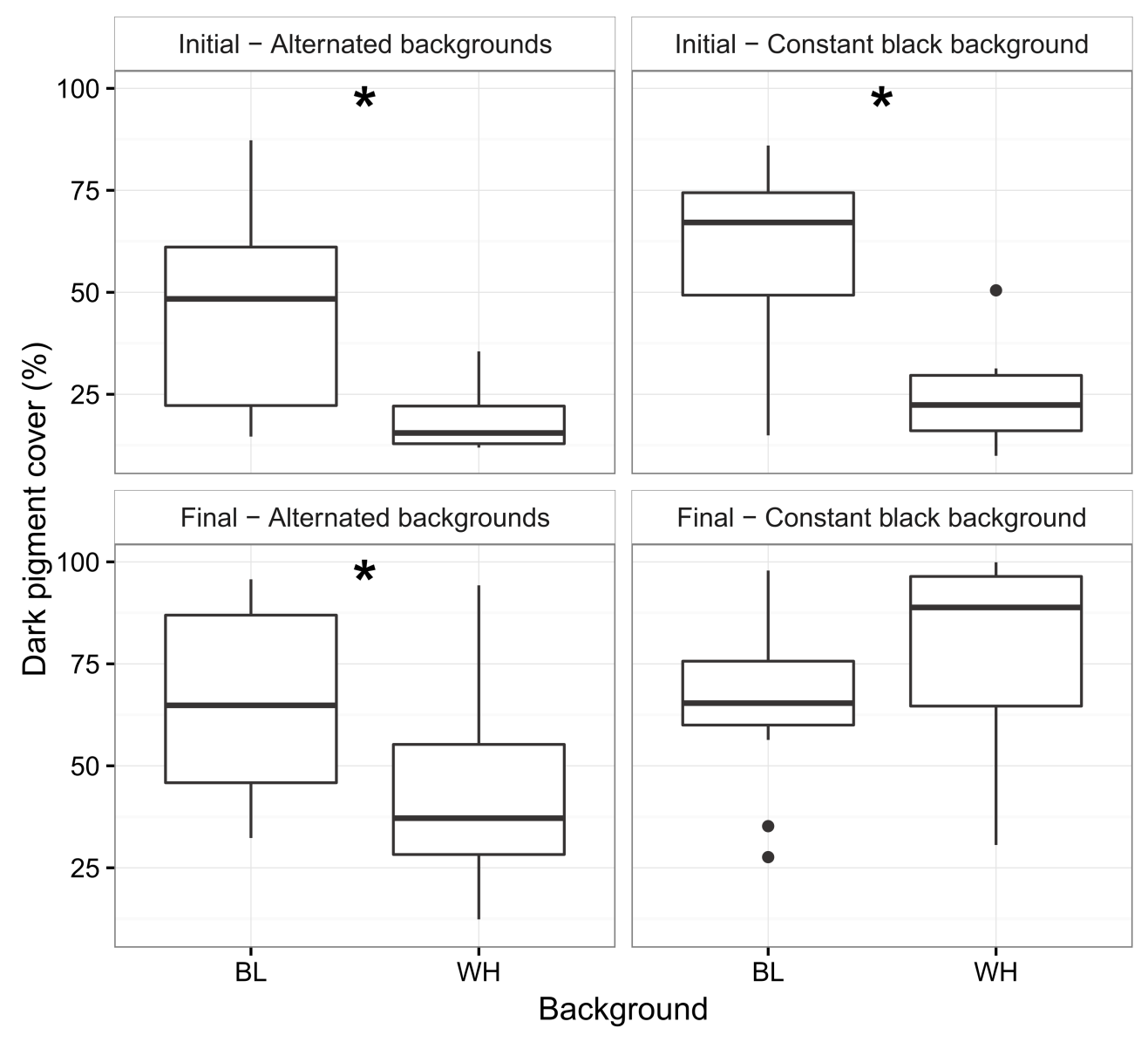


**Figure S4.** Box-and-whisker plots showing dark pigment cover (%) of *C. crangon* kept on constant black background (N = 11) or on alternating black and white backgrounds (N = 15) for 21 days. At day 0 (Initial) and day 21 (Final), shrimp were acclimated on a black (BL) background for 24h and moved to a white (WH) for 1 hour. *: P < 0.05 (Wilcoxon Signed Rank Test).
